# The Polymorphic AluYb8 Insertion in the *MUTYH* Gene is Associated with Reduced Type 1 Protein Expression and Reduced Mitochondrial DNA Content

**DOI:** 10.1371/journal.pone.0070718

**Published:** 2013-08-06

**Authors:** Wenwen Guo, Bixia Zheng, Zhenming Cai, Lizhi Xu, Dong Guo, Lili Cao, Yaping Wang

**Affiliations:** 1 Department of Medical Genetics, Nanjing University School of Medicine, Nanjing, China; 2 Jiangsu Key Laboratory of Molecular Medicine, Nanjing University School of Medicine, Nanjing, China; 3 The State Key Laboratory of Pharmaceutical Biotechnology, Nanjing University, Nanjing, China; Univeristy of California Riverside, United States of America

## Abstract

The human *mutY homolog* (*MUTYH*) participates in base excision repair (BER), which is critical for repairing oxidized DNA bases and maintaining DNA replication fidelity. The polymorphic AluYb8 insertion in the 15^th^ intron of the *MUTYH* gene (*AluYb8MUTYH*) has been shown to associate with an aggregated 8-hydroxy-2′-deoxyguanosine (8-OH-dG) lesion in genomic DNA and to serve as a risk factor for age-related diseases. In this work, we demonstrate that this variant is associated with a significant reduction of the type 1 MUTYH protein that localizes to mitochondria. Notably, this variant affects mitochondrial DNA (mtDNA) maintenance and functional mitochondrial mass in individuals homozygous for the *AluYb8MUTYH* variant. These findings provide evidence for an association between the *AluYb8MUTYH* variant and decreased mitochondrial homeostasis and, consequently, contribute to elucidating the roles of the *AluYb8MUTYH* variant in impairing the mitochondrial base excision repair (mtBER) system and increasing the risk of acquiring an age-related disease.

## Introduction

DNA damage is continuously caused by reactive oxygen species (ROS) generated from environmental exposure and normal cellular metabolism [1]. ROS are mainly produced in cells by the mitochondrial respiratory chain. Mitochondrial DNA (mtDNA) is vulnerable to ROS-induced oxidative damage due to its proximity to the respiratory chain and the lack of protective histones. Notably, 8-hydroxy-2′-deoxyguanosine (8-OHdG) is a major base lesion in DNA caused by ROS. This lesion is highly mutagenic because it readily mispairs with adenine residues, which leads to G:C to T:A transversions [2]. To avoid such mutagenesis, human cells have developed an efficient base excision repair (BER) system that is critical for repairing oxidized DNA and maintaining DNA replication fidelity. The human MutY glycosylase homolog (MUTYH, MIM 604933) participates in BER and increases replication fidelity by recognizing and removing misincorporated adenines opposite 8-OHdG during DNA replication [3]. Inherited defects in the *MUTYH* gene lead to an increased frequency of G:C to T:A mutations in the *adenomatous polyposis coli* (*APC*, MIM 611731) and *KRAS* (MIM 190070) genes [4]. Biallelic germline mutations in the *MUTYH* gene have been associated with high risk of adenomatous polyposis and cancer [5], [6]. Three types of primary transcriptions, α, β and γ-types mRNA transcriptions, with different 5′-untranslated regions were produced from three distinct exon 1 sequences in human *MUTYH* gene [7], but two major protein isoform (type 1 and type 2) were encoded by the *MUTYH* transcripts in human cells [8]. The type 1 MUTYH protein (CCDS41320.1), which harbors a mitochondrial targeting signal (MTS) at its N-terminus that encoded by MTS coding region of α-type mRNA transcription, is composed of 535 amino acids (calculated molecular mass, ∼60 kDa) and localizes to mitochondria. The type 2 MUTYH protein (CCDS41322.1) is composed of 521 amino acids (calculated molecular mass, ∼57 kDa) and localizes to nucleus because it lacks the MTS at its N-terminus.

**Figure 1:**
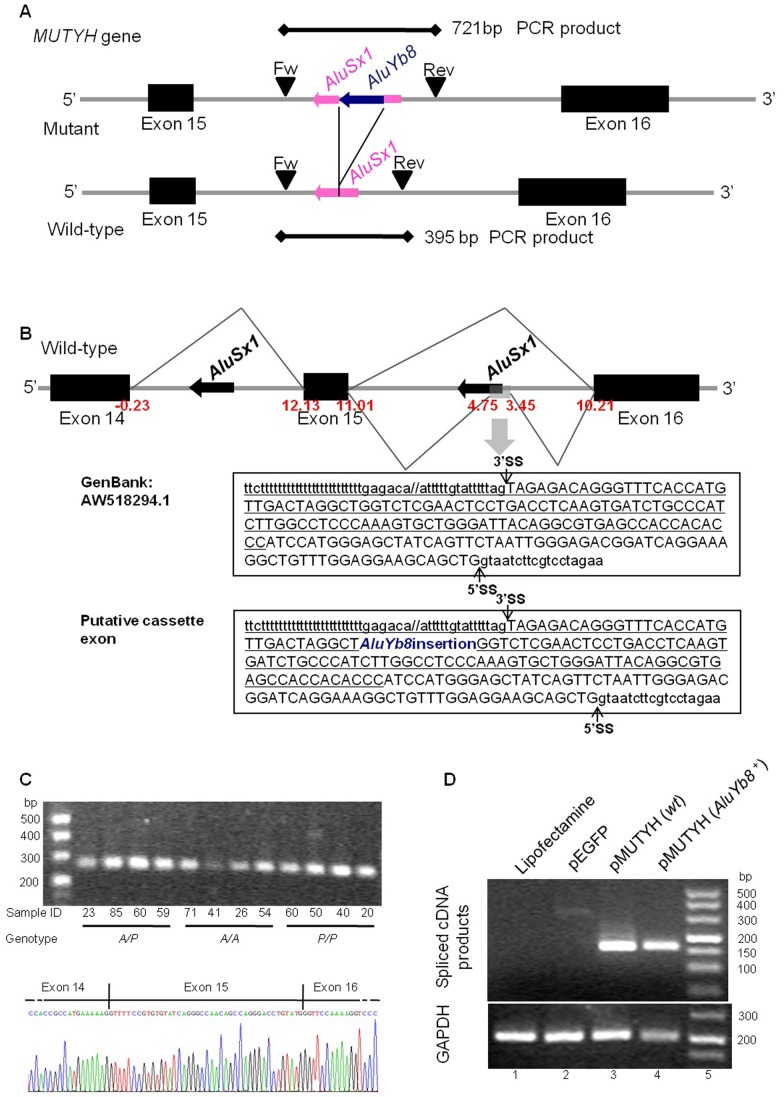
Illustration of the AluYb8 insertion in *MUTYH* gene and splicing analysis on this variant. (A) Schematic representation of the genomic structure of *MUTYH* from exon 15 to 16 indicates the position of the AluYb8 insertion in intron 15, depicted as a deep blue arrow. Exons are shown by boxes. The genotyping primers are represented by black arrowheads, and the lengths of their PCR products are also depicted. (B) A schematic illustration of the alternative splicing pattern between exons 14 and 16 of the *MUTYH* gene. Scores (MaxEnt score) of splice sites for the three exons were recorded in red. An alternatively spliced cassette exon within intron 15 was inferred from aligning two ESTs (BM679345.1 and AW518294.1) with human genomic DNA, and the putative cassette exon in the variant intron 15 is shown. (C) Splicing assays were performed in healthy adults. A representative ethidium bromide-stained agarose gel separating the RT-PCR products spanning *MUTYH* exon 14 to exon 16 are shown. PCR products were confirmed by sequencing (the bottom of the agarose gel electrophoresis). (D) The constructed wild-type (wt) and mutant minigene plasmids were transfected into 293 T cells, respectively, total RNA was extracted, and splicing products were separated on a 2% agarose gel after RT-PCR analysis. PCR products were sequenced. Similar results were obtained in two independent experiments.

We previously described an AluYb8 insertion in the 15^th^ intron of the *MUTYH* gene, *AluYb8MUTYH*, which is a common variant in Chinese population [9]. This variant was associated with the accumulation of ROS-induced oxidative lesions in DNA and with an increase in the cumulative risks for age-related diseases [9]. Further analysis demonstrated that *AluYb8MUTYH* heterozygous subjects were characteristic of the haploinsufficient model under conditions of oxidative stress and displayed more readily aggregated 8-OHdG lesions in their DNA [10]. Three *AluYb8MUTYH* genotypes are observed in the population, defined by the presence (*P*) or absence (*A*) of the AluYb8 insertion in each of the paired loci (Fig. 1A). These *AluYb8MUTYH* genotypes are categorized as homozygous absent (*A/A*), homozygous present (*P/P*) or heterozygous (*A/P*).

Alu elements are the most abundant family of short, interspersed repeat sequences in the human genome, with over 1.1 million copies. To date, several consequences of Alu insertions have been described [11], such as the introduction of novel *cis*-acting elements, modification of the promoter, and disruption of the reading frame or splicing pattern, yielding an alternatively spliced transcript. Recent findings suggest that a novel set of microRNA (miRNA) molecules were derived from Alu elements [12] and the Alu RNP, which composed of Alu RNA in complex with the signal recognition particle (SRP). The Alu RNA and Alu RNP do not affect localization but can regulate translation initiation [13], [14].

In the present study, we investigated how the *AluYb8MUTYH* variant affects *MUTYH* gene function. We analyzed *MUTYH* gene expression in *A/A*, *A/P* and *P/P* individuals. From MUTYH expression data, a reduced level of MUTYH expression was observed in individuals homozygous for the *AluYb8MUTYH* variant (*P/P*). Furthermore, we confirmed the altered MUTYH expression and its relevant biological effects using the cultured human fibroblast-like cells with three genotypes of the *MUTYH* gene.

## Materials and Methods

### Ethics Statement

All works on sample collection and processing, including the blood collection from adult participants, the collection of human umbilical cord blood and the umbilical cord sample from disconnected umbilical cords following full-term normal delivery, were carried out with the written informed consent from all of the recruited adults and the newborns’ parents. These works were approved by the ethics committee of Nanjing University School of Medicine.

### Study Participants

#### Participants set 1

Twelve healthy volunteers with the *A/A* genotype (mean age 34.3 years, age range 30 to 38 years old, 8 males and 4 females), seven healthy volunteers with the *P/P* genotype (mean age 32.1 years, age range 29 to 37 years old, 3 males and 4 females) and ten healthy volunteers with the *A/P* genotype (mean age 33.6 years, age range 24 to 43 years old, 9 males and 1 female) were recruited for blood collection into transcriptional analyses.

#### Participants set 2

Six healthy volunteers with the *A/A* genotype (mean age 27.8 years, age range 21 to 36 years old, 4 males and 2 females), six healthy volunteers with the *P/P* genotype (mean age 28.8 years, age range 22 to 35 years old, 4 males and 2 females) and nine healthy volunteers with the *A/P* genotype (mean age 25.0 years, age range 20 to 35 years old, 7 males and 2 females) participated in the MUTYH protein expression analysis with anti-MUTYH antibody (BS2535, Bioworld Technology, Inc.). Seven healthy volunteers (mean age 36.3 years, age range 25 to 46 years old, 5 males and 2 females) two for *A/A* genotype, one for *A/P* genotype and four for *P/P* genotype, were recruited for confirmatory experiment with a different MUTYH antibody that recognize a different epitope (sc-25169, Santa Cruz Biotechnology, Inc.) on the MUTYH protein expression.

#### Participants set 3

Eighteen healthy volunteers with the *A/A* genotype (mean age 32.8 years, age range 22 to 43 years old, 9 males and 9 females), eighteen healthy volunteers with the *P/P* genotype (mean age 34.1 years, age range 23 to 42 years old, 8 males and 10 females) and eighteen healthy volunteers with the *A/P* genotype (mean age 32.3 years, age range 22 to 44 years old, 8 males and 10 females) were recruited for blood collection into the study of nuclear and mitochondrial DNA damage by long-range quantitative PCR.

#### Participants set 4

Fifty-four newborns and 255 healthy adult volunteers were recruited for leukocyte mtDNA content detection. Human umbilical cord blood (HUCB) samples were obtained from disconnected umbilical cords of healthy individuals after birth following written informed consent from the newborns’ parents. Baseline participant characteristics in this section are presented in Table S1.

All of the recruited adults were nonsmokers and were free of clinical diseases, such as acute inflammation, tuberculosis, autoimmune diseases, cancer, cardiovascular diseases, diabetes and any neurological or psychiatric disorders, as assessed by medical history, physical examination and blood chemistry. The study was approved by the ethics committee of Nanjing University School of Medicine, and written informed consent was obtained from all of the recruited adults and the newborns’ parents.

### PCR-based Genotyping

The AluYb8 insertion in the intron 15^th^ of the *MUTYH* gene was detected by PCR as described previously [9].

### Minigene Construction and Transfection

For constructing the minigene plasmid, we amplified the wild-type and variant (defined by the AluYb8 insertion in intron 15) *MUTYH* fragments spanning exons 14, 15 and 16, from human genomic DNA from individuals with the *A/A* and *P/P* genotypes using oligonucleotide primers containing KpnI/BglII restriction sites. The fragments were digested by restriction enzymes and inserted between the KpnI/BglII sites in the pEGFP-C1 vector (Clontech) to obtain constructed minigene plasmids (pMUTYH-wt and pMUTYH-AluYb8^+^). All plasmids were confirmed by sequencing with an ABI PRISM 310 Genetic Analyzer and the Big Dye Terminator kit v3.0 (Applied Biosystems). For minigene insert sequences see Protocol S1.

The 293 T cells (human embryonic kidney epithelial cells, ATCC) were grown in Dulbecco’s Modified Eagle Medium (DMEM, 4.5 g/L glucose, GIBCO) supplemented with 10% fetal bovine serum (FBS, HyClone) and 1% penicillin-streptomycin (GIBCO), and cultured in 60-mm dishes under standard conditions at 37°C and 5% CO_2_. The recombinant minigene plasmid transfection was completed when the cultured cells were grown to 70% confluence, and then harvested after 48 h. To inhibit the nonsense-mediated decay of RNA, the transfected cells were treated with 300 µg/ml puromycin (Sigma) for 4 h before RNA extraction as described [15].

### Human Peripheral Blood Mononuclear Cells (PBMCs) Preparation

For immunoblot and transcriptional analysis, PBMCs were isolated by Ficoll Density Gradient (LTS1077, TBD Science) according to the manufacturer’s recommendations.

### RNA Isolation and Reverse Transcription (RT) –PCR

Total cellular RNA was extracted from isolated PBMCs and harvested cells using RNAiso Plus (Takara) followed by treatment with 2 U DNase I (RNase-free, Takara). Two micrograms of total RNA was transcribed with the All-in-One First-Strand cDNA Synthesis Kit according to the manufacturer’s instructions.

### Spliced cDNA Products Analysis

To analyze the effects of the *AluYb8MUTYH* variant on pre-mRNA splicing, PBMC cDNA was amplified with specific primers flanking intron 15 in *MUTYH*. The forward primer is located in exon 14 (Mutyh1635F), whereas the reverse primer is located in exon 16 (Mutyh1901R). Amplification was performed for 35 cycles, consisting of 30 sec at 94°C, 40 sec at 59°C, and 30 sec at 72°C.

The spliced cDNA products derived from the expressed minigenes in the transfected 293 T cells were detected by PCR with pEGFP-specific primer (Egfp1382R) and the exon 14 forward primer (Mutyh1691F). The following PCR conditions were used: 30 cycles of 30 sec at 94°C, 60 sec at 60°C, and 30 sec at 72°C. GAPDH (glyceraldehyde-3-phosphate dehydrogenase) mRNA levels were used as an internal control for each transfection.

PCR products were resolved on a 2% agarose gel and confirmed by sequencing. The PCR primer sequences are presented in Table S2.

### Transcription Analysis of the *MUTYH* Gene

The nucleotide sequences of all identified MUTYH transcripts [7] were retrieved from the NCBI (National Center for Biotechnology Information) database. We took α5 transcript (GenBank accession number NM_001128425.1), the longest known transcript of MUTYH, as reference sequence for primer design. For quantifying α-type *MUTYH* transcripts, we constructed a set of primers (Mutyh188F and Mutyh362R) from conserved sequences of α-type transcripts that yielded a PCR product spanning the region encoding MTS. To detect all *MUTYH* transcripts, a pair of primers (Mutyh1700F and Mutyh1901R) was used to amplify the coding sequences from exon 15 to exon 16 of the *MUTYH* gene.

All 29 healthy adult samples (Participants set 1) were used to determinate *MUTYH* transcription levels by quantitative real-time PCR (qrtPCR) with the designed primers using an ABI StepOne Real-Time System (Applied Biosystems) and Fast SYBR Green Master Mix (Applied Biosystems) as directed by the manufacturer. Each sample was tested in triplicate for each run, and all PCR runs were independently duplicated. The amplification efficiency of the fragment-of-interest was validated, and the melting curve analysis was used to verify non-specific products. The endogenous GAPDH mRNA levels were used for normalization. The primers and sequence information used for qrtPCR are presented in Table S2.

### Culture of Primary Umbilical Fibroblast-like Cells

Umbilical cords (n = 23; gestational ages, 39–40 weeks) were collected from healthy individuals after normal deliveries following written informed consent from their parents and approval from the ethics committee of Nanjing University School of Medicine. The tissues were processed immediately upon collection. The establishment of umbilical fibroblast-like cells from umbilical cord tissue of newborns and immunofluorescent staining of the specific molecular markers were performed as described [16]. The fibroblast-like cells were cultured in DMEM (1 g/L glucose, GIBCO) supplemented with 10% FBS (HyClone) and 1% penicillin-streptomycin (GIBCO) at 37°C and 5% CO_2_. When a homogeneous layer of fibroblast-like cells was observed, indicating that the culture was ready to passage. The first-passage cultures could be obtained by seeding the primary colonies as a homogeneous population of fibroblast-like cells (Fig. S5A).

### Isolation of Mitochondria

Mitochondrial fractions were prepared from cultured fibroblast-like cells (passage number 4) using a Mitochondria Isolation Kit for Cultured Cells (Thermo Scientific) according to the manufacturer’s instructions.

### Cell/mitochondria Lysis and Immunoblot Assays

Whole cell extract was prepared using Nonidet P-40 lysis buffer (1% (v/v) NP-40, 50 mM Tris-HCl pH 8.0, 150 mM NaCl, 1 mM phenylmethylsulfonyl fluoride), and mitochondrial extract was prepared using 2% CHAPS (25 mM Tris, 150 mM NaCl, 2% (v/v) CHAPS, pH 7.2). Both preparations were performed in the presence of a 1 × protease inhibitor cocktail solution (Amresco) on ice for 15 min and were clarified by centrifugation (12,000 *g* for 15 min, 4°C).

Proteins were separated on 12% polyacrylamide gels and transferred to PVDF membranes (0.45 μΜ, Millipore). The membranes were probed with the specific primary antibodies (see Protocol S2) followed by the appropriate horseradish peroxidase-conjugated secondary antibodies. The signal was detected using a chemiluminescent HRP substrate (Millipore).

### Long-range Quantitative PCR Assay

Long-range quantitative PCR (long-range qPCR) assays were performed to assess the nuclear and mitochondrial base excision repair capacity in healthy adults (Participants set 3), and the procedure is modified slightly as described previously [17]. Basically, total DNA was extracted from whole blood using the TIANamp Genomic DNA Kit (Tiangen Biotech. Co. Ltd.). Initial DNA concentration of each sample was measured using PicoGreen double-stranded DNA binding agent (Invitrogen) and DNA standard curve generated by serial diluted λ/HindIII DNA. Each DNA sample was diluted with TE, pH 7.4 to 3 ng per microliter. Primer pairs for a 13.5-kb fragment from *β-globin* loci and for an 8,843-bp fragment from mtDNA were used for calculating nDNA and mtDNA integrity, respectively, and were indirectly reflected the functional condition of the nuclear and mitochondrial base excision repair, respectively. Primer sequences are listed in Table S2. The long-range qPCR reaction was performed with TaKaRa LA Taq PCR kit (Takara) as follows: 15 ng total DNA, in a reaction mix of 50 µl, with 1 × buffer, dNTPs at 200 µM for each nucleotide, 2.5 mM final concentration of Mg^2+^, 20 pmol for each of primer and 1 unit Taq DNA polymerase. Thirty PCR cycles were used for quantitative amplification of 13.5-kb fragment from β-globin loci, and twenty-six PCR cycles were used for quantitative amplification of 8,843-bp fragment from mtDNA. PCR products were separated on 0.8% agarose gels to exclude that non-specific products had been amplified. 50%-control (half of the amount of the test DNA template) and blank-control (no-template DNA) were taken as quality control and blank value, respectively. PCR products were quantified using fluorescence measurements by the PicoGreen double-stranded DNA binding agent (Invitrogen). PCR products of long fragment from mtDNA were normalized to the amplification of a 108-bp mtDNA fragment to account for the effect of mtDNA copy number on long-range PCR amplification. These normalized fluorescence values were used to calculate the relative amplification by comparison between the amplification of target samples and the mean value from the *A/A* group. At least two independent experiments were performed for each sample.

### mtDNA Content Quantification and Fluorescence-based Quantitative PCR (FQ-PCR)

We quantified mtDNA by FQ-PCR using FastStart Universal SYBR Green Master Mix (Roche) with a StepOne Real-Time System (Applied Biosystems). Genomic DNA was used as a template, which was extracted from blood by the TIANamp Genomic DNA Kit (Tiangen Biotech. Co. Ltd.), and was amplified with specific primer sets (Table S2), where the selected mtDNA fragments reflected the mtDNA content and the β-actin sequence reflected the nuclear DNA (nDNA) content. Real-time PCR was employed to determine the relative mtDNA content by estimating the threshold cycle number of the mitochondrial fragment and of the nuclear gene fragment. The real-time PCR was performed in triplicate for each sample. All PCR products were resolved by melting curve analysis and electrophoresis on a 2% agarose gel to exclude non-specific amplification.

### Mitochondrial Mass Assay and Oxygen Consumption Measurement

The fibroblast-like cells with the three genotypes of *MUTYH* gene were cultured in DMEM (4.5 g/L glucose, GIBCO) supplemented with 10% FBS (HyClone), and were passed in parallel and expanded in culture. The respiring mitochondrial mass in the cultured fibroblast-like cells (passage number 5) was determined by fluorescence levels upon staining with MitoTracker Red (Molecular Probes) using the BD FACSAria cell sorter (BD Biosciences). For each measurement, 10,000 events were counted. The data were analyzed using FlowJo software (Tree Star).

Homozygous mutant (*P/P*) and wild-type (*A/A*) cells (passage number 5) were harvested with trypsin and pooled in a total volume of 2.5 ml of the cell suspension, and 2.0 ml of the cell suspension was immediately added to the oxygen electrode chamber for assay of basal oxygen consumption rate. The oxygen consumption rate of the cultured fibroblast-like cells was measured using the Oxytherm System from Hansatech Instruments (Norfolk, United Kingdom) according to the manufacturer’s instructions. Cell counts were performed via hemocytometer to assure that equivalent cell numbers were utilized for all measurements.

### Confocal Microscopy and Image Processing

The fibroblast-like cells (passage number 5) were cultured on glass cover slips, and the respiring mitochondrial intensity was determined by the MitoTracker Red fluorescence using a FV1000 confocal microscope (Olympus). The results from the three *AluYb8MUTYH* genotypes were compared with independent experiment and were processed in parallel. Fluorescence was quantified with ImageJ software (NIH), and the integrated pixel density for cells was determined with a uniform threshold.

### Bioinformatic Analysis

Alu subfamilies (modern dimeric Alu repeats and its monomeric ancestors), the insertion orientation, and the borders of the *MUTYH* genomic sequence were determined using the RepeatMasker (Smit, AFA and Green, P. RepeatMasker at http://repeatmasker.org/). Splice site strengths for 5′ and 3′ sites were determined by maximum entropy scores using consensus splice site models in MAXENT (ME5’ss and ME3’ss, respectively) [18].

### Statistical Analysis

All statistical analyses were performed using the SPSS statistical package version 16.0 (SPSS Inc.). Separate variable comparisons among subjects with different *AluYb8MUTYH* genotypes were conducted by the nonparametric Kruskal-Wallis test or multinomial logistic regression. Logarithmic transformation of data was used for the multinomial logistic regression analysis, because the original values of the mtDNA content showed a skewed distribution (Fig. S1, the original data of mtDNA content distribution). Gender distribution among genotype categories was estimated with the chi-square test. For analysis of statistical difference between experiments involving cultured fibroblast-like cells, a 2-sided t-test was applied. *P*<0.05 was considered statistically significant.

## Results

### *AluYb8MUTYH* did not Influence Precursor mRNA Processing Event

Alu elements are conserved, ∼280-nucleotide-long repeat sequences that reside mostly in introns, 3′ untranslated regions and intergenic genomic regions [19]. Intronic Alu insertions are transcribed with the host gene as part of precursor mRNAs (pre-mRNAs). The transcribed Alu RNAs, embedded into pre-mRNAs, were in fact functionally involved in pre-mRNA processing events and could influence the splicing of flanking exons, particularly with Alu sequences in the reverse direction [20]. Therefore, we examined the distribution of Alu elements in the *MUTYH* gene (Fig. S2).

The *AluYb8MUTYH* variant is characterized by a reverse AluYb8 sequence inserted at the left arm of an AluSx1 sequence, located 34-bp away from the AT-rich linker (A_5_TACA_6_), which originally existed in the 15^th^ intron of *MUTYH*. We accessed a set of clustered Expressed Sequences (EST/mRNA) (UniGene Hs.271353, MUTYH) and identified two alternative splicing events, BM679345.1 and AW518294.1, that harbor a cassette exon containing the integrant Alu repetitive element belong to the intrinsic AluSx1 in the 15^th^ intron of *MUTYH* (Fig. 1B). Therefore, we checked the possible alternative splicing events of the exon-skipping or Alu exonization derived from the Alu (AluSx1 and AluYb8) insertion in the intron 15. We performed the analysis of their splicing patterns in leukocytes using reverse transcription/PCR with specific primers spanning exon 14 to exon 16 within the *MUTYH* gene (Primers in Table S2). However, we failed to identify any abnormal splicing pattern containing novel splice variants regardless of the *AluYb8MUTYH* genotypes (Fig. 1C). The similar result was observed from a minigene splicing reporter system for the *MUTYH* gene (Fig. 1D), which was utilized to study the splicing pattern of the cassette and flanking exons in mammalian cell lines.

### Transcriptional Analysis in PBMCs

**Figure 2:**
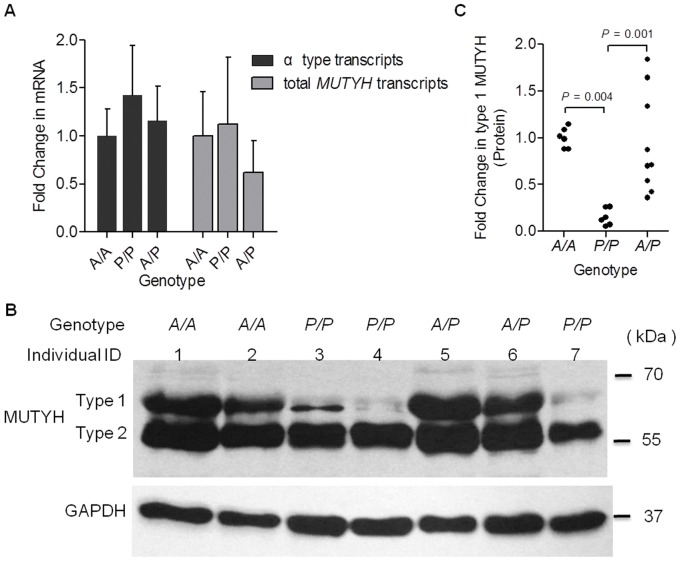
Analysis of the association between the *AluYb8MUTYH* and MUTYH expression. (A) Transcriptional analysis by qrtPCR of *MUTYH* targets in PBMCs from healthy adult individuals. The abundance of *MUTYH* targeted transcripts normalized to *GAPDH* gene are shown, and the values are expressed as fold changes relative to mean mRNA levels of each type of targeted transcripts in the *A/A* genotype group. Error bars indicate the standard error of the mean. (B) Representative immunoblot for anti-MUTYH antibody (BS2535, Bioworld Technology, Inc.) showing the protein expression of MUTYH and GAPDH in PBMCs whole cell extracts from seven healthy adult individuals. This panel shows the altered pattern of MUTYH expression in PBMCs from the homozygous *P/P* individuals. The two major MUTYH types, type 1 and type 2, are indicated. GAPDH was used as a protein loading control. The individual IDs are shown. (C) MUTYH quantification. Densitometric quantification of the bands was performed with Image J software (NIH) and normalized to the GAPDH signal. Type 1 MUTYH protein levels are expressed as fold values relative to GAPDH expression. Solid circles represent the checked individuals in the protein expression analysis. *P*-values are indicated, by Kruskal-Wallis test.

We detected the abundance of two sets of MUTYH mRNA transcripts (α-type transcripts and total *MUTYH* transcripts) in PBMCs isolated from recruited well-documented healthy adults (n = 29, Participants set 1) by qrtPCR using sequence-specific primer pairs. We observed a similar transcriptional abundance of the α-type *MUTYH* mRNA transcripts, which harbor the MTS coding region, among the individuals grouped by the three *AluYb8MUTYH* genotypes (Fig. 2A). Analogous results were obtained for mRNA expression assay of the total *MUTYH* transcripts (Fig. 2A).

### A substantial Change of the MUTYH Protein Expression Associated with *AluYb8MUTYH*

**Figure 3:**
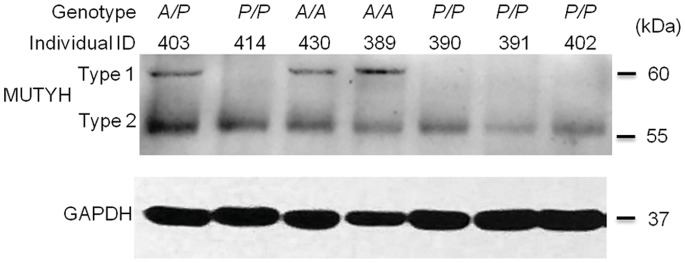
MUTYH protein expression detected by immunoblot analysis with MUTYH antibody (sc-25169). The two major MUTYH proteins, type 1 and type 2, are indicated. GAPDH was used as a protein loading control. The *AluYb8MUTYH* genotypes and individual IDs are shown, respectively.

Immunoblot analysis performed with whole cell extracts of PBMCs from healthy adults (Participants set 2) by anti-MUTYH antibody (BS2535, Bioworld Technology, Inc.) demonstrated an altered MUTYH expression in the *P/P* genotype. Here, we investigated two major MUTYH proteins in PBMCs, type 1 and type 2, which migrate at an apparent molecular mass of 60 kDa and 57 kDa, respectively (Fig. 2B and Fig. S3). The type 1 MUTYH protein was significantly reduced by approximately 82% and 81% in PBMCs from healthy young donors with the *P/P* genotype (n = 6) when compared to healthy young donors with the *A/A* (n = 6) and *A/P* (n = 9) genotypes, respectively (Fig. 2C; *P* = 0.004 for *A/A* versus *P/P* and *P* = 0.001 for *A/P* versus *P/P*, by Kruskal-Wallis test). To confirm this result, we conducted immunoblot analysis using a different MUTYH antibody (sc-25169, Santa Cruz Biotechnology, Inc.) that recognize a different epitope mapping near the N-terminus of MUTYH protein in whole cell extracts of PBMCs from seven randomly selected healthy subjects (four of them with *P/P* genotype). The result of confirmation experiment also showed the expression of type 1 MUTYH protein were substantial reduction in the healthy individuals with the *P/P* genotype (Fig. 3).

In addition, the expression of type 2 MUTYH protein in the *P/P* donors was mildly decreased when compared with that in the *A/A* donors (Fig. S4A, *P* = 0.01 by Kruskal-Wallis test). Thus, the ratio of expression levels between the type 1 and type 2 MUTYH protein (MUTYH expression pattern) is markedly decreased in the PBMCs homozygous for *AluYb8MUTYH* versus PBMCs with *A/A* or *A/P* genotypes (Fig. S4B; *P* = 0.01 for *P/P* versus *A/A* and *P* = 0.005 for *P/P* versus *A/P*, by Kruskal-Wallis test).

### The Expression of Type 1 MUTYH Protein was Greatly Reduced in Mitochondria of the Cultured Fibroblast-like Cells with *P/P* Genotype

The characteristic phenotypes of the primary fibroblast-like cells were detected with the specific molecular markers, α-SMA (alpha smooth muscle actin), vimentin and type I collagen, by immunofluorescent staining. The fibroblast-like cells showed positive staining for α-SMA (Fig. S5B), and staining for vimentin (Fig. S5C) and type I collagen (Fig. S5D), similar to the previous reported [16].

**Figure 4:**
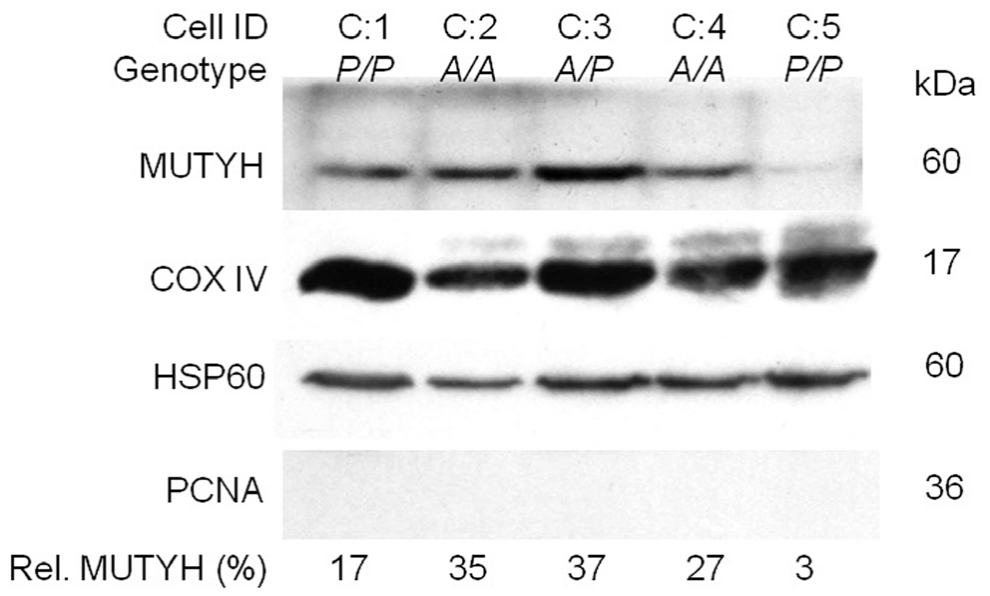
Expression of type 1 MUTYH protein in the mitochondria of cultured fibroblast-like cells. Mitochondrial fractions were prepared from cultured fibroblast-like cells (passage number 4). The genotypes of the cultured cells and their corresponding cell IDs are indicated. The MUTYH signal for anti-MUTYH antibody (BS2535) was normalized to COX IV loading control by Image J software (NIH), and the mitochondrial MUTYH percentage (Rel. MUTYH) is indicated at the bottom of each column. COX IV, cytochrome c oxidase subunit IV. HSP 60, heat shock protein 60. PCNA, proliferating cell nuclear antigen.

To confirm the result that the reduced expression of type 1 MUTYH protein in PBMCs was associated with *AluYb8MUTYH* variant, the primary cultured fibroblast-like cells with *A/A*, *A/P* and *P/P* genotypes were established from newborn umbilical cord tissue. We evaluated the levels of type 1 MUTYH protein with COX IV (loading control) that localized to the mitochondria in isolated mitochondrial extracts from the cultured fibroblast-like cells (passage number 4) with different *AluYb8MUTYH* genotypes by immunoblot analysis. Our results showed that the type 1 MUTYH protein was greatly reduced in mitochondrial extracts from *P/P* cells (Fig. 4 and Fig. S6).

### *AluYb8MUTYH* Affected the Base-excision Repair of MUTYH Glycosylase in Mitochondria

MUTYH protein is one type of A/G-specific adenine DNA glycosylase participating in starting of base excision repair by recognizing 8-oxodG:A mismatch and removing the misincorporated adenine opposite 8-oxodG, leaving a processing repair intermediate, an apurinic/apyrimidinic site (AP site). The resulting AP sites can block the progression of the polymerase resulting in decreased amplification of a target sequences during PCR assay [21], although the resulting AP site is further processed by AP endonucleases, DNA polymerase, and ligase in human cells. The reduction of MUTYH protein would limit the repair of damaged bases, and indicated less resulting AP sites remaining in DNA. Herein, we performed a long-range PCR to identify whether the altered MUTYH protein expression would change the amplification efficiency of long genomic targets in nDNA or mtDNA.

**Figure 5:**
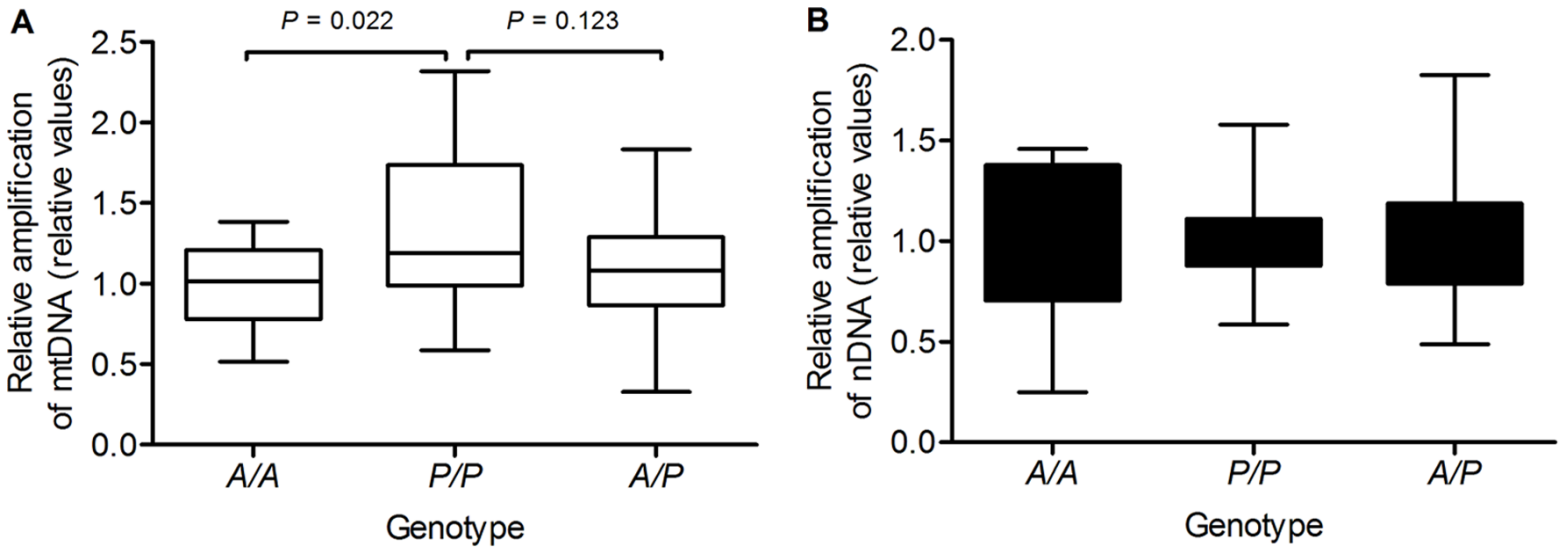
The relative amplification of long-range targets among the cells with different *AluYb8MUTYH* genotypes. The long-range PCR was performed for both the mitochondrial DNA (A, open bars) and nuclear β-globin fragments (B, solid bars) among the healthy individuals aged 22 to 44 with different *AluYb8MUTYH* genotypes. The boxes cover the 25^th^ to 75^th^ percentiles, and the minimal and maximal values are shown by the ends of the bars. Relative amplification is presented relative to average of *A/A* group values, n = 18. *P*-values are indicated, multinomial logistic regression.

As shown in Figure 5, long-range PCR assay of genomic DNA from *A/A* (n = 18), *P/P* (n = 18) and *A/P* (n = 18) cases, the relative PCR amplification of nDNA were similar among the three genotype groups (1.00±0.38, 1.01±0.25 and 1.02±0.31, respectively, mean ± SD). However, the relative amplification of mtDNA was elevated significantly (*P* = 0.022, multinomial logistic regression analysis) in *P/P* cases (1.30±0.45, mean ± SD) than that in *A/A* cases (1.00±0.26, mean ± SD). The relative amplification of mtDNA in the *P/P* cases was also higher when compared with *A/P* cases (1.08±0.35, mean ± SD), but the difference between the two groups was not significant (*P* = 0.123, multinomial logistic regression analysis). These results implied that the reduced expression of type 1 MUTYH protein in PBMCs with the *P/P* genotype could result in less remaining of processing AP sites in mtDNA during oxidative DNA damage repair.

### *AluYb8MUTYH* Associated with Reduced Leukocyte mtDNA Content

**Figure 6:**
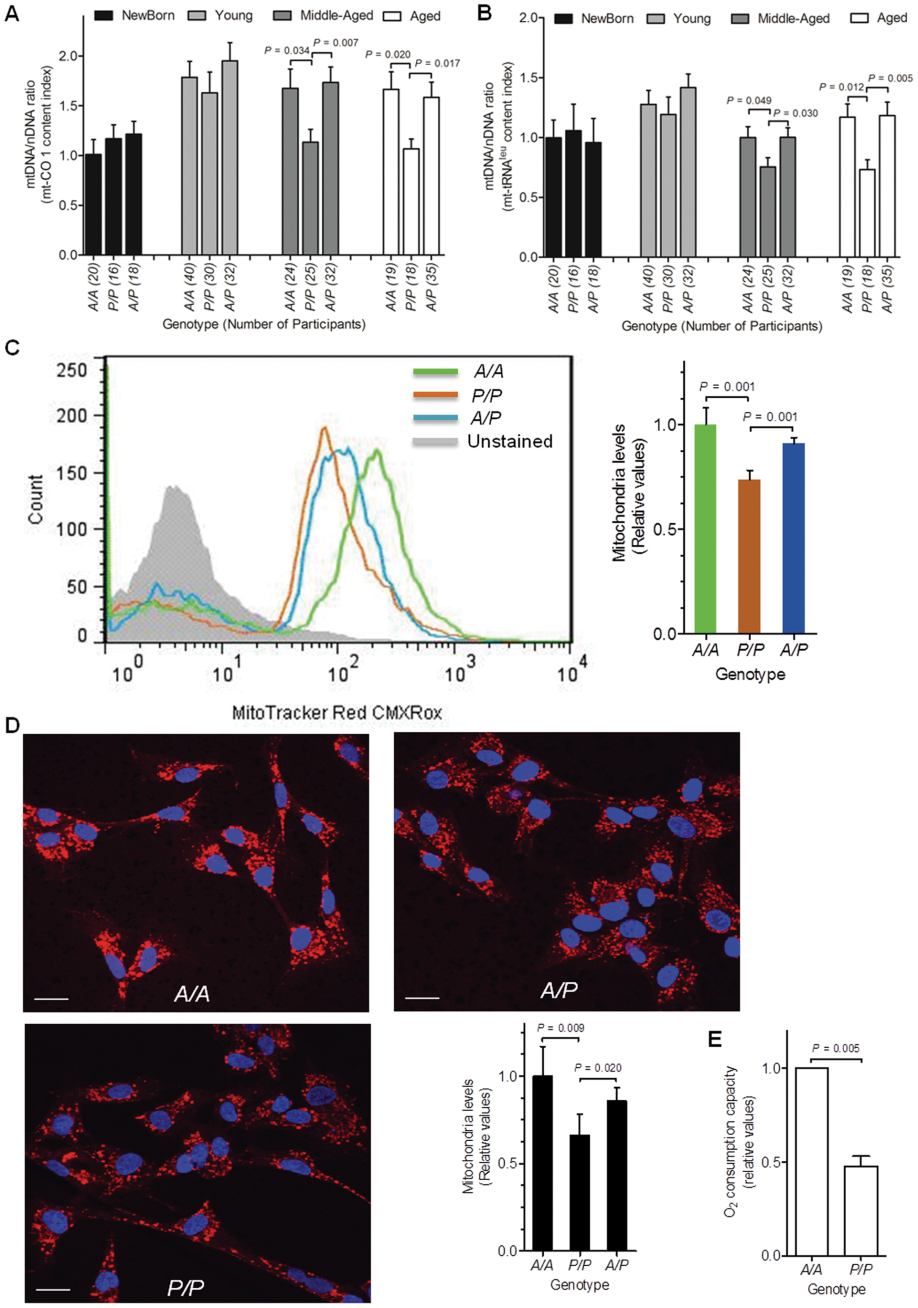
The alteration of mitochondria phenotypes associated with the *AluYb8MUTYH*. (A, B) The leukocytic mtDNA content was associated with the *AluYb8MUTYH* variant. The relative amounts of mt-CO 1 and mt-tRNA^Leu^ content in the newborn, young, middle-aged and aged groups are shown. The mean mtDNA/nDNA ratio in the *A/A* genotype group of healthy newborn infants was set to 1. Error bars indicate the standard error of the mean. The number of participants is shown in brackets. *P*-values are indicated (multinomial logistic regression). (C) The respiring mitochondria mass in cultured cells (passage number 5) of C:2, C:3 and C:5 was detected by flow cytometry. The results were confirmed in triplicate, and similar results were obtained when other cultured fibroblast-like cells with different *AluYb8MUTYH* genotypes were used. Error bars represent the mean ± SD with n = 3. The geometric mean value of fluorescence intensity in the *A/A* cells was set to 1. *P*-values are indicated (2-sided t-test). (D) The respiring mitochondria mass were confirmed in cultured cells by confocal microscopy. Representative images from independent experiments are shown. DAPI (4′, 6-diamidino-2-phenylin-dole, Sigma) nuclear staining is shown. Scale bar, 20 µm. Five images of each fibroblast-like cell culture, with the indicated genotype, from the same experiment were quantified. Fluorescence was quantified with Image J software (NIH). A plot of the relative values of the respiring mitochondria levels is displayed in the lower right corner. The geometric mean of the fluorescence intensity in the *A/A* cells was set to 1. The values are presented as the mean ± SD (n = 5). *P*-values are indicated (2-sided t-test). The result was confirmed in triplicate. (E) Relative oxygen consumption capacity of intact cells (*P/P* and *A/A* cells). The oxygen consumption rate in *A/A* cells was set to 1. The data are the mean ± SD from at least two independent experiments. *P*-value is indicated (2-sided t-test).

To assess mtDNA content we chose two small regions in the mitochondrial genome (a 197 bp fragment and a 108 bp fragment) as mtDNA copy number indexes, mt-CO 1 index (mitochondria-encoded cytochrome c oxidase subunit 1) and mt-tRNA^Leu^ index (mitochondria-encoded tRNA leucine 1), respectively, due to the low probability of introducing lesions in small segments. The relative mtDNA content was analyzed using FQ-PCR by calculating the mtDNA/nDNA ratio by normalizing each mitochondrial fluorescence amplification measurement to the corresponding β-actin signal in blood samples. These investigated individuals (Participants set 4) was separately analyzed in the newborn and adult groups. Notably, we observed a similar mtDNA/nDNA level (both indexes) among all three genotype groups in newborn infants (Fig. S7A and B). Moreover, in the adults, the *A* allele carriers (with *A/A* and *A/P* genotypes) displayed significantly higher levels of mtDNA content versus the carriers homozygous for *P* allele (Fig. S7A and B). Considering the influence of age in mtDNA content, we repeated the analysis with the age stratification: newborn, younger (20 to 44 years old), middle-aged (45 to 59 years old), and aged (60 and older) groups. The mt-CO 1 content index was significantly decreased in the *P/P* genotype group compared with the *A/A* or *A/P* genotype groups in the middle-aged and older population (*P* = 0.034 and *P* = 0.007 for *A/A* versus *P/P* and *A/P* versus *P/P*, respectively, in middle-aged group; *P* = 0.020 and *P* = 0.017 for *A/A* versus *P/P* and *A/P* versus *P/P*, respectively, in older group) (Fig. 6A). Likewise, the mt-tRNA^Leu^ content index was also significantly decreased in the *P/P* genotype group compared with the *A/A* or *A/P* genotype groups in the middle-aged and older population (*P* = 0.049 and *P* = 0.030 for *A/A* versus *P/P* and *A/P* versus *P/P*, respectively, in middle-aged group; *P* = 0.012 and *P* = 0.005 for *A/A* versus *P/P* and *A/P* versus *P/P*, respectively, in older group) (Fig. 6B).

### *AluYb8MUTYH* Changed the Respiring Mitochondria Levels and Basal Oxygen Respiration Rate of the Cultured Fibroblast-like Cells

We used primary cultured umbilical fibroblast-like cells to compare the respiring mitochondria mass and basal oxygen consumption rate among the three genotypes of *MUTYH* gene. Flow cytometric analysis showed more respiring mitochondria in *A/A* and *A/P* cells than that in *P/P* cells (*P* = 0.001 and *P* = 0.001 for *A/A* versus *P/P* and *A/P* versus *P/P*, respectively, by 2-sided t-test) (Fig. 6C), and the similar results were obtained by confocal microscopy analyses (*P* = 0.009 and *P* = 0.020 for *A/A* versus *P/P* and *A/P* versus *P/P*, respectively, by 2-sided t-test) (Fig. 6D). We also tested the basal whole cell oxygen consumption rate of the homozygous mutant (*P/P*) and wild-type (*A/A*) cells. As shown in Figure 6E, the relative oxygen respiration rate was lower in the homozygous mutant cells than in the wild-type cells (*P* = 0.005, by 2-sided t-test).

## Discussion

A typical Alu element contains multiple potential splice donor and acceptor sites [22], which are involved in Alu exonization and alternative splicing under certain conditions [23]. Currently, 238,000 Alu elements are estimated to reside within introns of protein-coding genes, and some of them may affect the splicing pattern of flanking exons [24]. In the present study, we first considered whether *AluYb8MUTYH* could alter the splicing pattern of the flanking exons in the *MUTYH* gene. We screened the mature *MUTYH* mRNAs in leukocytes from healthy individuals and in the constructed minigene-transfected cells. However, the AluYb8 insertion in the *MUTYH* gene did not observably influence precursor mRNA processing events.

Previous studies have reported that multiple authentic isoforms, which localized to either nucleus or mitochondria, were identified as mammalian homolog of MutY DNA glycosylase by immunoblot analysis in cultured cells [25]–[27], brain [28], thymus [29] and liver tissues [30]. However, here we investigated the indicated migration of the major MUTYH isoforms corresponding to the expected sizes of type 1 (60 kDa) and type 2 (57 kDa) in PBMCs (Fig. 2B, Fig. 3 and Fig. S3), that are similar to in vitro-translated MUTYH with the transcription/translation-coupled system [8], [31]. We considered whether the AluYb8 insertion in the 15^th^ intron of *MUTYH* gene affected the expression of MUTYH protein. The immunoblot assays showed the *AluYb8MUTYH* could cause a substantial change in the expression of MUTYH protein. The type 1 MUTYH protein was significantly reduced in PBMCs from the individuals with homozygous *AluYb8MUTYH* (*P/P* genotype) when compared to the individuals with homozygous wild-type (*P* = 0.004 by Kruskal-Wallis test) or heterozygous (*P* = 0.001 by Kruskal-Wallis test) (Fig. 2C). We then performed two additional experiments to validate this result of the immunoblot assays: the primary cultured umbilical fibroblast-like cells from the individuals with *P/P*, *A/P* or *A/A* genotypes were used to isolate mitochondrial fraction and to detect mitochondrial MUTYH protein; a different antibody that recognize a different epitope (mapping near the N-terminus of MUTYH protein) was applied to detect MUTYH protein of PBMCs among health individuals with the three genotypes. Both of the experiments confirmed the AluYb8 insertion in 15^th^ intron of *MUTYH* was associated with an inhibition of the type 1 MUTYH protein expression, and the reduction of the type 1 MUTYH protein in *P/P* individuals shifted the MUTYH expression pattern (type 1/type 2) to a lower ratio. We quantified the mRNA expression of the *MUTYH* gene (the α-type transcripts and the total *MUTYH* transcripts) in the PBMCs of healthy adults and did not observe differences in MUTYH transcript levels among the three *AluYb8MUTYH* genotypes (Fig. 2A). In this work, we detected both of the common isoforms (approximately 57 kDa and 60 kDa) in PBMCs by immunoblot analysis (Fig. 2B, Fig. 3 and Fig. S3). Ohtsubo et al. reported that human *MUTYH* gene was expressed in more than ten alternatively spliced transcripts encoding at least seven isoforms of the MUTYH protein [25]. The other protein isoforms encoded by *MUTYH* transcript variance were not apparent in this work either due to a lower level of expression or the expression only occurs in specific tissues, such as the 47-kDa-MUTYH isoform observed in brain tissue [28].

The mitochondrial BER pathway has recently been thoroughly described [32] and is a valuable pathway for repairing ROS-induced mtDNA damage from endogenous and exogenous sources. This pathway relies on excision of damaged bases by glycosylase and AP endonuclease. The type 1 MUTYH protein localizes in mitochondria and represents one type of A/G-specific adenine DNA glycosylase, initiating the mitochondrial BER pathway to ensuring correct and efficient repair of 8-oxodG lesions in mtDNA. The decrease of the type 1 MUTYH protein would injure mtDNA repair capacity. The long-range qPCR assay was performed to assess their proficiency for mtDNA and nDNA repair in these cells with different *AluYb8MUTYH* genotypes. Lower levels of relative PCR amplification of mtDNA were observed in these cells with *A/A* and *A/P* genotypes than in cells homozygous for *AluYb8MUTYH* variant (*P/P*) (Fig. 5A), whereas the level of the relative PCR amplification of nDNA was unaffected (Fig. 5B). The well-documented repair process of the MUTYH glycosylase in BER pathway had suggested that reduced type 1 MUTYH protein expression would decrease the efficiency of damaged base excision in mitochondria [32], and is associated with the progression of the polymerase during long-range PCR, which could result in a change in amplification of target sequences.

In mammalian cells, reliable mtDNA replication and mtDNA integrity are necessary for mtDNA maintenance, although the regulation of mtDNA maintenance is extremely complex, including mitochondrial DNA polymerase (pol γ), transcriptional cofactors and the stimulus environment [33]. Similar levels of relative mtDNA content indexes (mt-CO 1 and mt-tRNA^leu^) were observed among the three *AluYb8MUTYH* genotypes in infants, but decreased levels of relative mtDNA content indexes (mt-CO 1 and mt-tRNA^leu^) were apparent in middle-aged and older subjects homozygous for *AluYb8MUTYH* variant (*P/P*) in comparison with wild-type (*A/A*) and heterozygote (*A/P*) groups when we categorized the adult subjects based on age: younger, middle-aged, and aged (Fig. 6A and B). Those results indicated that mtDNA content differences among the three genotype of the *AluYb8MUTYH* variant manifested at the population level due to a progressive accumulation of damaged DNA bases with ageing. This processing of the progressive accumulation may reduce the rate of mtDNA turnover.

Normally, mtDNA levels appear to correlate to organelle number [34]. We observed that the cultured *A/A* and *A/P* cells had relatively more respiring mitochondria than *P/P* cells (Fig. 6C and D). Oxygen consumption measurements demonstrated a reduced basal oxygen consumption rate in the cultured cells homozygous for the *AluYb8MUTYH* allele than in the wild-type cells (Fig. 6E). It is consistent with the observed reduction of the mitochondrial mass in the homozygous mutant cells.

To date, Alu element insertions have contributed to a significant proportion of human genetic diseases [19] and participate in the regulation of affected genes. Recently, a novel set of small RNA molecules derived from the non-coding elements transcribed by the RNA polymerase III were identified in genome [12]. Among these non-coding elements, Alu repetitive sequence was one of the most investigated. The Alu elements can be transcribed by internal RNA polymerase III promoter, particularly the youngest Alu subfamilies (AluY subfamilies). Alu RNA can bind the cognate signal recognition particle (SRP) protein to form Alu ribonucleoprotein (Alu RNP). The transcribed Alu RNA and thereby forming complex, Alu RNP, could have a important role in regulation of translation initiation [13]. Hasler and Strub constructed Alu RNPs composed of Alu RNA bound to SRP9/14, and observed the roles of the constructed Alu RNP in protein translation in vitro system [13]. They found that Alu RNP can inhibit protein translation at the level of initiation [13]. In present study, we did not determine any abnormal splicing derived from the intronic AluYb8 insertion in *MUTYH* gene. However, we found the *AluYb8MUTYH* variant was associated with an altered *MUTYH* expression at the post-transcriptional level, primarily on the regulation of protein translation for the type 1 MUTYH protein. Although the precise mechanism for the reduced expression of type 1 protein correlated with the intronic AluYb8 insertion in *MUTYH* gene remains to be clarified, it is reasonable to consider that the regulatory mechanism of Alu RNA and Alu RNP on protein translation could also exists in *AluYb8MUTYH* mediated the expression inhibition of type 1 protein.

The type 1 MUTYH protein in PBMCs have a relatively constant expression level in homozygous carriers, either in *A/A* or *P/P* genotypes, although a wide variation of the protein expression level was observed in *A/P* heterozygotes (Fig. 2C). In addition, a variation in type 1 MUTYH protein expression was shown in two cultured fibroblast-like cells with the *P/P* genotype (C:1 and C:5 in Figure 4). These results indicate that the expression of MUTYH protein could depend on cell type or the cell oxidative stress status, and the heterozygous cells (*A/P* genotype) would exhibit a haploinsufficiency under condition of oxidative stress. Functional investigation indicated that the AluYb8 insertion in the 15^th^ intron of *MUTYH* gene associated with the reduction of the type 1 MUTYH protein could impair the mtDNA repair capacity. The decreased mtDNA content and the respiring mitochondria mass in the cells with *P/P* genotype could make the cells in a reduced basal oxygen respiration rate, thereby affecting the reserve capacity of cells counteract oxidative stress. Although the *AluYb8MUTYH*-homozygous subjects recruited for this study were still healthy, they would have a higher risk in the conditions of oxidative stress, which are likely associated with aging and age-related diseases [35]. On the fact that about one-fifth of the Chinese population are homozygous for the *AluYb8MUTYH* [9], this variant may represent one of the key targets for mechanisms that participate in aging and age-related diseases.

## Supporting Information

Figure S1 **The original data of mtDNA content distribution (mt-CO 1 content and mt-tRNA^Leu^ indexes) for each age group.**
(TIF)

Figure S2 **Schematic illustration of Alu elements’ distribution in the** ***MUTYH*** **gene.** The Alu elements are illustrated with black and blue arrows, where the indicated orientations of the Alu are relative to the pre-mRNA. The position and transcriptional direction of the alternative first exons (1α, 1β and 1λ) are indicated.
(TIF)

Figure S3 **The MUTYH protein expression detected in whole cell extracts by immunoblot assay using anti-MUTYH antibody (BS2535, Bioworld Technology, Inc.).** This panel shows the altered pattern of MUTYH expression in the homozygous *P/P* individuals. The two major MUTYH proteins, type 1 and type 2, are indicated. GAPDH was used as a protein loading control. The *AluYb8MUTYH* genotypes and individual IDs are shown, respectively.
(TIF)

Figure S4 **MUTYH protein quantification was assessed by immunoblot analysis in PBMCs from healthy subjects using anti-MUTYH antibody (BS2535, Bioworld Technology, Inc.).** (A) Type 2 MUTYH protein levels are expressed as fold values relative to GAPDH expression. (B) MUTYH type 1/type 2 ratios are plotted. Solid circles represent the checked individuals analyzed by immunoblot. Densitometric quantification of the bands was performed with Image J software (NIH) and normalized to GAPDH. *P*-values are indicated, by Kruskal-Wallis test.
(TIF)

Figure S5 **Morphological characterization and immunofluorescent staining of the primary cultured umbilical fibroblast-like cells.** (A) A representative microphotograph of umbilical fibroblast-like cells at first passage. (B-D) The cultured fibroblast-like cells (passage number 3) stain positive for α-SMA (B), and for vimentin (C) and type I collagen (D). DAPI-stained nuclei. Scale bar, 200 µm. The presented results were obtained from one primary cultured cell line with the *A/A* genotype. Similar morphology and immunofluorescent staining is observed for all cultured fibroblast-like cell populations regardless of the *AluYb8MUTYH* genotype.
(TIF)

Figure S6 **Expression of the type 1 MUTYH protein in the mitochondrial fractions from three cultured fibroblast-like cells with different** ***AluYb8MUTYH*** **genotypes (passage number 4).** Immunoblot assay was performed with anti-MUTYH antibody (BS2535, Bioworld Technology, Inc.) and anti-COX IV antibody, which used as a loading control. The checked membrane was re-probed with the mitochondrial (HSP60) and the nuclear (PCNA) marker antibodies to assess the fractions purity. The genotypes of the cultured cells and their corresponding cell IDs are indicated. COX IV, cytochrome c oxidase subunit IV; HSP 60, heat shock protein 60; PCNA, proliferating cell nuclear antigen.
(TIF)

Figure S7 **The analysis of associated between reduced leukocytic mtDNA content and the** ***AluYb8MUTYH*** **.** (A, B) The relative amounts of mt-CO 1 content index (A) and mt-tRNA^Leu^ content index (B) in newborn and adult groups are shown. The relative mtDNA content of *A/A* groups in newborn infants was set to 1. Error bars indicate the standard error of the mean. *P*-values are indicated, by Kruskal-Wallis test. The number of participants is shown in brackets.
(TIF)

Table S1 **Sample profile included in survey population for the investigation of leukocyte mtDNA content: demographic by genotype and age group.**
(PDF)

Table S2 **Primer sequences.**
(PDF)

Protocol S1 ***MUTYH*** **minigene sequences.**
(PDF)

Protocol S2 **Antibodies.**
(PDF)
