# Supplementary figures and images for: The Polymorphic AluYb8 Insertion in the MUTYH Gene is Associated with Reduced Type 1 Protein Expression and Reduced Mitochondrial DNA Content
Source: PLoS One. 2013 Aug 6;8(8):e70718. doi: 10.1371/journal.pone.0070718 (PMC3735632; doi:10.1371/journal.pone.0070718)

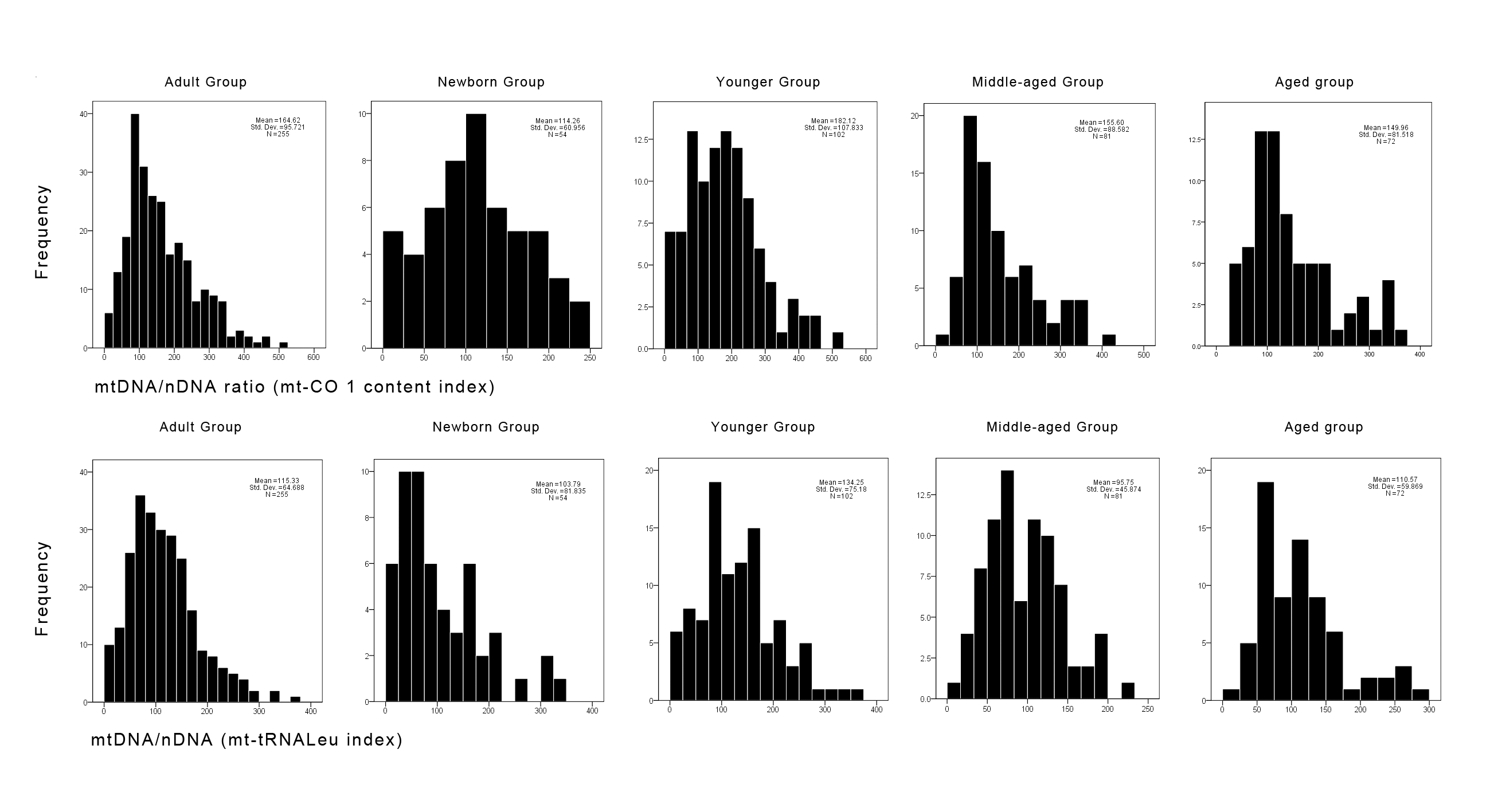

Supplement: Figure S1 — The original data of mtDNA content distribution (mt-CO 1 content and mt-tRNALeu indexes) for each age group. (TIF) [file pone.0070718.s001.tif]

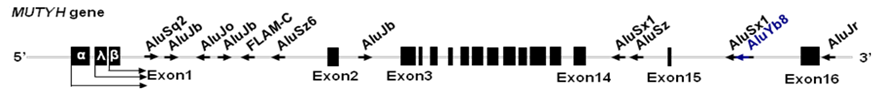

Supplement: Figure S2 — Schematic illustration of Alu elements’ distribution in the MUTYH gene. The Alu elements are illustrated with black and blue arrows, where the indicated orientations of the Alu are relative to the pre-mRNA. The position and transcriptional direction of the alternative first exons (1α, 1β and 1λ) are indicated. (TIF) [file pone.0070718.s002.tif]

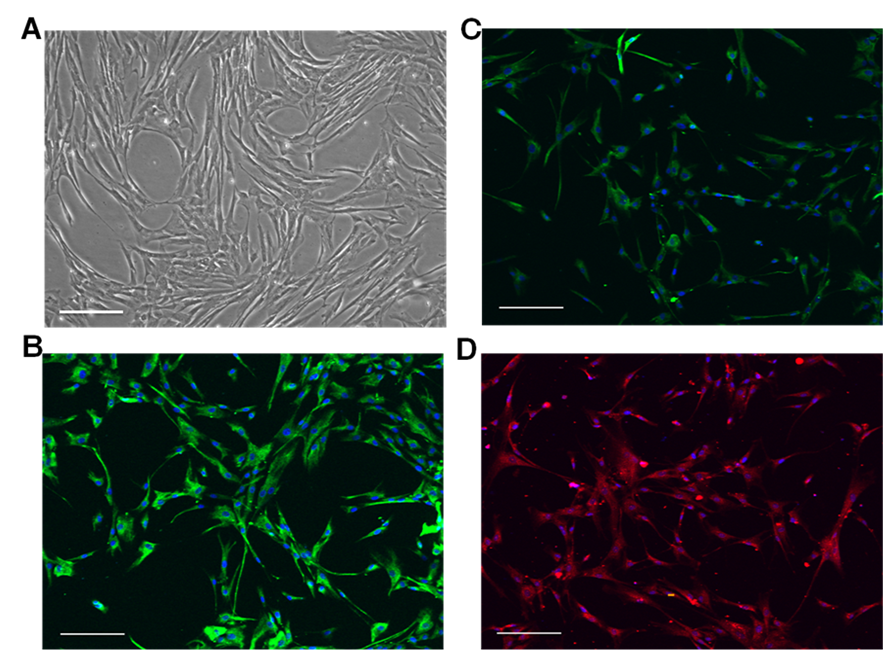

Supplement: Figure S5 — Morphological characterization and immunofluorescent staining of the primary cultured umbilical fibroblast-like cells. (A) A representative microphotograph of umbilical fibroblast-like cells at first passage. (B-D) The cultured fibroblast-like cells (passage number 3) stain positive for α-SMA (B), and for vimentin (C) and type I collagen (D). DAPI-stained nuclei. Scale bar, 200 µm. The presented results were obtained from one primary cultured cell line with the A/A genotype. Similar morphology and immunofluorescent staining is observed for all cultured fibroblast-like cell populations regardless of the AluYb8MUTYH genotype. (TIF) [file pone.0070718.s005.tif]

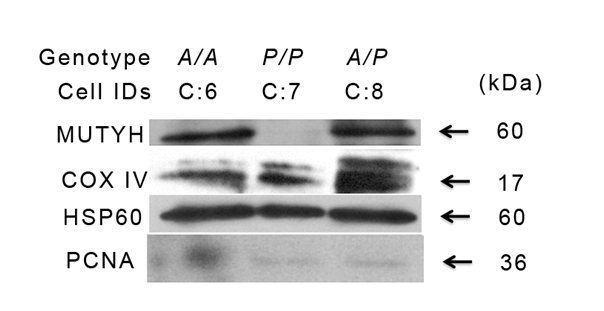

Supplement: Figure S6 — Expression of the type 1 MUTYH protein in the mitochondrial fractions from three cultured fibroblast-like cells with different AluYb8MUTYH genotypes (passage number 4). Immunoblot assay was performed with anti-MUTYH antibody (BS2535, Bioworld Technology, Inc.) and anti-COX IV antibody, which used as a loading control. The checked membrane was re-probed with the mitochondrial (HSP60) and the nuclear (PCNA) marker antibodies to assess the fractions purity. The genotypes of the cultured cells and their corresponding cell IDs are indicated. COX IV, cytochrome c oxidase subunit IV; HSP 60, heat shock protein 60; PCNA, proliferating cell nuclear antigen. (TIF) [file pone.0070718.s006.tif]
