# Supplementary material for: The Polymorphic AluYb8 Insertion in the MUTYH Gene is Associated with Reduced Type 1 Protein Expression and Reduced Mitochondrial DNA Content
Source: PLoS One. 2013 Aug 6;8(8):e70718. doi: 10.1371/journal.pone.0070718 (PMC3735632; doi:10.1371/journal.pone.0070718)
Supplement: Table S1 — Sample profile included in survey population for the investigation of leukocyte mtDNA content: demographic by genotype and age group. (PDF) [file pone.0070718.s008.pdf]

Table S1. Sample profile included in survey population for the investigation of leukocyte mtDNA content: demographic by genotype and age group.

Description by	Genotype Categories of *AluYb8MUTYH*			*P*-value
Age Group	*A/A*	*P/P*	*A/P*	
Age (years, Mean ± SD)				
NewBorn				
Young	30.8 ± 7.62	27.8 ± 6.65	29.4 ± 6.72	0.222
Middle-Aged	50.4 ± 4.68	48.6 ± 4.13	48.7 ± 3.77	0.243
Aged	67.1 ± 6.78	66.6 ± 6.52	69.0 ± 5.79	0.234
Number of Participants				
NewBorn	20	16	18	
Young	40	30	32	
Middle-Aged	24	25	32	
Aged	19	18	35	
Gender (female/male)				
NewBorn	7/13	5/11	7/11	0.897
Young	21/19	19/11	20/12	0.580
Middle-Aged	10/14	13/12	15/17	0.769
Aged	6/13	5/13	11/24	0.957
