# Supplementary material for: The Polymorphic AluYb8 Insertion in the MUTYH Gene is Associated with Reduced Type 1 Protein Expression and Reduced Mitochondrial DNA Content
Source: PLoS One. 2013 Aug 6;8(8):e70718. doi: 10.1371/journal.pone.0070718 (PMC3735632; doi:10.1371/journal.pone.0070718)
Supplement: Table S2 — Primer sequences. (PDF) [file pone.0070718.s009.pdf]

Table S2. Primer sequences.

**Oligo Name**	**Sequence(5' to 3')**	**RefSeq Accession**
Primers used for genotyping		
Mutyh15182F	GAGTTTTGTGGGATATGAATTGTGG	NG_008189.1
Mutyh15576R	CTTCCTCCAAACAGCCTTTCCT	NG_008189.1
Primers used for minigene construction		
pMutyh14066F	TGAAGATCTGAATAGCCAAGGATGTTGGCT	NG_008189.1
pMutyh16130R	GCAGGTACCTGGAGATGTGAGACCGAAAG	NG_008189.1
Primers used for the spliced cDNA products analysis		
Mutyh1635F	TCGCTGGCTGACGCAGGAGGAA	NM_001128425.1
Mutyh1901R	ACAGGATTCTCAGGGAATGG	NM_001128425.1
Mutyh1691F	AGGTTTTCCGTGTGTATCAGGGC	NM_001128425.1
Egfp1382R	GCGGTACCGTCGACTGCAGAAT	U55763.1
GAPDH192F	TGTTGCCATCAATGACCCCTT	NM_002046.3
GAPDH393R	CTCCACGACGTACTCAGCG	NM_002046.3
Primers used for *MUTYH* transcript level analysis		
Mutyh188F	AAACTGCGCCATCGTCACTG	NM_001128425.1
Mutyh362R	GAAGGCTTGGCCTGACTGTTG	NM_001128425.1

Table S2. Primer Sequences. | Continued

**Oligo Name**	**Sequence(5' to 3')**	**RefSeq Accession**
Mutyh1700F	GTGTGTATCAGGGCCAACAG	NM_001128425.1
Mutyh 1901R	ACAGGATTCTCAGGGAATGG	NM_001128425.1
GAPDH534F	CAAGATCATCAGCAATGCCT	NM_002046.3
GAPDH626R	ATGAGTCCTTCCACGATACC	NM_002046.3
Primers used for long-range quantitative RCP assay		
β-globin48510F	CGAGTAAGAGACCATTGTGGCAG	U01317.1
β-globin61989R	GCACTGGCTTAGGAGTTGGACT	U01317.1
mt5999F	TCTAAGCCTCCTTATTCGAGCCGA	NC_012920.1
mt14841R	TTTCATCATGCGGAGATGTTGGATGG	NC_012920.1
Primers used for mitochondrial DNA content quantification		
mt-CO1_F	TTCGCCGACCGTTGACTATTCTCT	NC_012920.1
mt-CO1_R	AAGATTATTACAAATGCATGGGC	NC_012920.1
mt-tRNA^{Leu1}_F	CACCCAAGAACAGGGTTTGT	NC_012920.1
mt-tRNA^{Leu1}_R	TGGCCATGGGTATGTTGTTAA	NC_012920.1
β-Actin6995F	TCACCCACACTGTGCCCATCTACGA	NG_007992.1
β-Actin7289R	CAGCGGAACCGCTCATTGCCAATGG	NG_007992.1

F, Forward primer. R, Reverse primer. RefSeq Accession, GenBank accession number.

pMutyh14066F and pMutyh16130R, the recognition sequences of restriction enzymes are underlined.
