# Supplementary material for: The Polymorphic AluYb8 Insertion in the MUTYH Gene is Associated with Reduced Type 1 Protein Expression and Reduced Mitochondrial DNA Content
Source: PLoS One. 2013 Aug 6;8(8):e70718. doi: 10.1371/journal.pone.0070718 (PMC3735632; doi:10.1371/journal.pone.0070718)
Supplement: Protocol S1 — MUTYH minigene sequences. (PDF) [file pone.0070718.s010.pdf]

Protocol S1

MUTYH minigene sequences

Exons are shown in yellow. The intrinsic AluSx1 of intron 15 is marked by green, and AluYb8 insertion in intron 15 is underlined.

The corresponding minigene sequences are as follows:

MUTYH-wt minigene sequence

5'GAATAGCCAAGGATGTTGGCTTTTGAGGCTATATCCACAGGCCTATTTGAACCCCT
TGACCCTTCCTCCAGGTTGTCCACACCTTCTCTCACATCAAGCTGACATATCAAGTAT
ATGGGCTGGCCTTGGAAGGGCAGACCCCAGTGACCACCGTACCACCAGGTGCTCGC
TGGCTGACGCAGGAGGAATTTCACACCGCAGCTGTTTCCACCGCCATGAAAAAGGC
ACTACCTTTGTTGTCTTTGTTGTACTTCCTTGTGTTTCCTACATGTTCTACATGAATAT
ATTACTGTGTAAACAGGAAAAAAAGCATTTTTTTTTGAGACGGAGAATCGCTCTGTT
GCCCAGGCTGGAGTGCAATGGCGCTATCTCGGTTGACTGCAACCTCCATCTCCCGGG
TTCAAGTGATTCTCCTGCCTCAGCTTCCTGAGTAGCTCGGATTACAGGCGCCCGCCA
CCATGCCTGGCTAATTTTTGTATTTTTAGTAGAGATGAGGTTTCACCATGCTGGCCAG
GCTGGTCTCCAACTCTTGACCTCAAGTGATCCGCCCGCCTCAGCCTCCCAGAATGCT
GGGATTACAGGTGTGAGCTACCACACCCAGCCATGATTTTTTGTATTTTTAGAGATG
GGGTTTCACCATGTTGGCCAGGCTGGTCTCAAACTCCTGGCCTCAAGTGATCCACCC
GACTTGGCCTCCCAAAATGCTGGGATTATAGGCGTGAGCCACCATGCCTGGCCAAA
AAAGCATATTTTAAACAAAAGTACTGGGACATGAAGTTAAGGGCAGAACACCGGTT
TATCTCTTTTGCAAAAAGTGCCAGCCCTCACCTCCCTGTCTTCTTGTCTAGGTTTTCC

GTGTGTATCAGGGCCAACAGCCAGGGACCTGTATGGTAAGTCTCCTAGGCCTCTCCC
AACCGTGTCTCCCCAGGCCTGAGTCCATAGGTTTTAGTCAGTTAACTAACGAATGT
CTGGGTGAACATTCTCCACTCCAGGCTTCACTGGAGGGAGGAATAGTTCTTGACCTG
GAGACCTTCCATTGTGGGGTGCAGGGTAGAGGGAAAGGAAAAAAATGATGAGGACT
CTCCAGTGTCACAGGTGTGTGAAATGCCTGTACTGAGTTTTGTGGGATATGAATTGT
GGAGCCATCAGTTCTTTTTTTTTTTTTTTTTTTTTTTTGAGACAGTCTCACTGTCACCC
AAGCTAGAGTGCAGTGGCCCGATCTCGGCTCACTGCAATCTCCCACAACTGGATTAA
AGCGATTCTCCTGCCTCAGTCTCCCAAGTAGCTGGGATTACAGGTGCCTGCTACCAC
ACCCAACTAATTTTTGTATTTTTAGTAGAGACAGGGTTTCACCATGTTGACTAGGCTG
GTCTCGAACTCCTGACCTCAAGTGATCTGCCCATCTTGGCCTCCCAAAGTGCTGGGA
TTACAGGCGTGAGCCACCACACCCATCCATGGGAGCTATCAGTTCTAATTGGGAGAC
GGATCAGGAAAGGCTGTTTGGAGGAAGCAGCTGGTAATCTTCGTCCTAGAAATGAA
TCCTTTTCTTCAGGTTTGGGAGGGGAATCAACCGTAGCGATGTTCTTTCCAGTCCCAA
GAGTAGTGTGAGCAAAGGTGGAGCCCAGTGCAGGCTGCAGTGCTCAAGGAGCTGTG
AGCAGTTGGTTTAGCTGGAAAGGTCAGCAGGGCCTGCTGGGTCAGTCAGGGTGTGA
GCCCTAGGCTGCTGACTCATTTTAGGTGGAGGAGAGAGGAGTCAGATTTGCATTTTT
AGGACAATTCGATTCCCTCAGGCAGCCTTTTGGAGGGTTGATTGATGGGGCAGATAC
TTGAGGCAGGATGAAAGCTCTACAGCATTCCAGGCTAAGCCTAGCTAGATCAGTAG
AGTCGGGGAAAGGGAGAGAGGACAAGGAGAGGATTCTCTGCTCCCCCTCCCCCAAC
TACAAGGCCTCCCTCCTTCCATTTTTTCACAGGGTTCCAAAAGGTCCCAGGTGTCCTC
TCCGTGCAGTCGGAAAAAGCCCCGCATGGGCCAGCAAGTCCTGGATAATTTCTTTCG

GTCTCACATCTCCA$^{3'}$

MUTYH-AluYb8^{+} minigene sequence

$^{5'}$GAATAGCCAAGGATGTTGGCTTTTGAGGCTATATCCACAGGCCTATTTGAACCCCT

TGACCCTTCCTCCAGGTTGTCCACACCTTCTCTCACATCAAGCTGACATATCAAGTAT

ATGGGCTGGCCTTGGAAGGGCAGACCCCAGTGACCACCGTACCACCAGGTGCTCGC

TGGCTGACGCAGGAGGAATTTCACACCGCAGCTGTTTCCACCGCCATGAAAAAGGC

ACTACCTTTGTTGTCTTTGTTGTACTTCCTTGTGTTTCCTACATGTTCTACATGAATAT

ATTACTGTGTAAACAGGAAAAAAAGCATTTTTTTTTGAGACGGAGAATCGCTCTGTT

GCCCAGGCTGGAGTGCAATGGCGCTATCTCGGTTGACTGCAACCTCCATCTCCCGGG

TTCAAGTGATTCTCCTGCCTCAGCTTCCTGAGTAGCTCGGATTACAGGCGCCCGCCA

CCATGCCTGGCTAATTTTTGTATTTTTAGTAGAGATGAGGTTTCACCATGCTGGCCAG

GCTGGTCTCCAACTCTTGACCTCAAGTGATCCGCCCGCCTCAGCCTCCCAGAATGCT

GGGATTACAGGTGTGAGCTACCACACCCAGCCATGATTTTTTGTATTTTTAGAGATG

GGGTTTCACCATGTTGGCCAGGCTGGTCTCAAACTCCTGGCCTCAAGTGATCCACCC

GACTTGGCCTCCCAAAATGCTGGGATTATAGGCGTGAGCCACCATGCCTGGCCAAA

AAAGCATATTTTAAACAAAAGTACTGGGACATGAAGTTAAGGGCAGAACACCGGTT

TATCTCTTTTGCAAAAAGTGCCAGCCCTCACCTCCCTGTCTTCTTGTCTAGGTTTTCC

GTGTGTATCAGGGCCAACAGCCAGGGACCTGTATGGTAAGTCTCCTAGGCCTCTCCC

AACCGTGTCTCCCCAGGCCTGAGTCCATAGGTTTTTAGTCAGTTAACTAACGAATGT

CTGGGTGAACATTCTCCACTCCAGGCTTCACTGGAGGGAGGAATAGTTCTTGACCTG

GAGACCTTCCATTGTGGGGTGCAGGGTAGAGGGAAAGGAAAAAAATGATGAGGACT

CTCCAGTGTCACAGGTGTGTGAAATGCCTGTACTGAGTTTTGTGGGATATGAATTGT

GGAGCCATCAGTTCTTTTTTTTTTTTTTTTTTTTTTTTGAGACAGTCTCACTGTCACCC

AAGCTAGAGTGCAGTGGCCCGATCTCGGCTCACTGCAATCTCCCACAACTGGATTAA

AGCGATTCTCCTGCCTCAGTCTCCCAAGTAGCTGGGATTACAGGTGCCTGCTACCAC

ACCCAACTAATTTTTGTATTTTTAGTAGAGACAGGGTTTCACCATGTTGACTAGGCT*G*

*GTCTCGAACTCC*TTTTTTTTTTTTTTTTTTTTTTTTGAGACGGAGTCTCGCTCTGTCGGC

CCAGGCCAGACTGCGGACTGCAGTGGCGCAATCTCGGCTCACTGCAAGCTCCGCTTC

CCGGGTTCACGCCATTCTCCTGCCTCAGCCTCCCGAGTAGCTGGGACTACAGGCGCC

CGCCACCGTGCCCGGCTAATTTTTTGTATTTTTAGTAGAGACGGGGTTTCCCCTTGTTA

GCCAGGATGGTCTCGATCTCCTGACCTCATGATCCACCCGCCTCGGCCTCCCAAAGGC

TGGGATTACAGGCGTGAGCCACCGCGCCCGGCCCGGTCTCGAACTCCTGACCTCAAG

TGATCTGCCCATCTTGGCCTCCCAAAGTGCTGGGATTACAGGCGTGAGCCACCACAC

CCATCCATGGGAGCTATCAGTTCTAATTGGGAGACGGATCAGGAAAGGCTGTTTGG

AGGAAGCAGCTGGTAATCTTCGTCCTAGAAATGAATCCTTTTCTTCAGGTTTGGGAG

GGGAATCAACCGTAGCGATGTTCTTTCCAGTCCCAAGAGTAGTGTGAGCAAAGGTG

GAGCCCAGTGCAGGCTGCAGTGCTCAAGGAGCTGTGAGCAGTTGGTTTAGCTGGAA

AGGTCAGCAGGGCCTGCTGGGTCAGTCAGGGTGTGAGCCCTAGGCTGCTGACTCATT

TTAGGTGGAGGAGAGAGGAGTCAGATTTGCATTTTTAGGACAATTCGATTCCCTCAG

GCAGCCTTTTGGAGGGTTGATTGATGGGGCAGATACTTGAGGCAGGATGAAAGCTC

TACAGCATTCCAGGCTAAGCCTAGCTAGATCAGTAGAGTCGGGGAAAGGGAGAGAG

GACAAGGAGAGGATTCTCTGCTCCCCTCCCCCAACTACAAGGCCTCCCTCCTTCCA

TTTTTTCACAGGGTTCCAAAAGGTCCCAGGTGTCCTCTCCGTGCAGTCGGAAAAAGC

CCCGCATGGGCCAGCAAGTCCTGGATAATTTCTTTCGGTCTCACATCTCCA3’
