# Supplementary material for: The Polymorphic AluYb8 Insertion in the MUTYH Gene is Associated with Reduced Type 1 Protein Expression and Reduced Mitochondrial DNA Content
Source: PLoS One. 2013 Aug 6;8(8):e70718. doi: 10.1371/journal.pone.0070718 (PMC3735632; doi:10.1371/journal.pone.0070718)
Supplement: Protocol S2 — Antibodies. (PDF) [file pone.0070718.s011.pdf]

Protocol S2

Antibodies

The antibodies used in immunofluorescent staining of cultured fibroblast-like cells are as follows: Primary antibodies, α-SMA antibody (ab5694, Abcam), vimentin (BM0135, BOSTER) and Collagen α1 Type I (COL1A1, sc-8784, Santa cruz). For secondary antibodies, goat anti-rabbit IgG-FITC (sc-2012, Santa cruz), goat anti-mouse IgG-FITC (sc-2010, Santa cruz) and donkey anti-goat IgG (H+L)-Cy3 (A0502, Beyotime) antibodies were used.

The antibodies used in immunoblotting as follows: Primary antibodies, anti-MUTYH (BS2535, Bioworld Technology, Inc.), anti-MUTYH (sc-25169, Santa Cruz Biotechnology, Inc.), anti-cytochrome c oxidase subunit IV (anti-COX IV, ab14744, Abcam), anti-glyceraldehyde 3-phosphate dehydrogenase (anti-GAPDH, AG019, Beyotime), anti-heat shock protein 60 (anti-HSP 60, BS1179, Bioworld Technology, Inc.) and anti-proliferating cell nuclear antigen (anti-PCNA, BS1289, Bioworld Technology, Inc.) antibodies. For secondary antibodies, HRP Conjugated Goat anti-Rabbit IgG (#21002, Abmart) and HRP Conjugated Goat anti-Mouse IgG (H+L) (A0216, Beyotime) were used.
